# A Systematic Review of Recent Advances in Equine Influenza Vaccination

**DOI:** 10.3390/vaccines2040797

**Published:** 2014-11-14

**Authors:** Romain Paillot

**Affiliations:** Animal Health Trust, Centre for Preventive Medicine, Lanwades Park, Newmarket, Suffolk CB8 7UU, UK; E-Mail: romain.paillot@aht.org.uk; Tel.: +44-1638-751000 (ext. 1208); Fax: +44-1638-555634

**Keywords:** equine influenza, vaccine, equid

## Abstract

Equine influenza (EI) is a major respiratory disease of horses, which is still causing substantial outbreaks worldwide despite several decades of surveillance and prevention. Alongside quarantine procedures, vaccination is widely used to prevent or limit spread of the disease. The panel of EI vaccines commercially available is probably one of the most varied, including whole inactivated virus vaccines, Immuno-Stimulating Complex adjuvanted vaccines (ISCOM and ISCOM-Matrix), a live attenuated equine influenza virus (EIV) vaccine and a recombinant poxvirus-vectored vaccine. Several other strategies of vaccination are also evaluated. This systematic review reports the advances of EI vaccines during the last few years as well as some of the mechanisms behind the inefficient or sub-optimal response of horses to vaccination.

## 1. Introduction 

### 1.1. Introduction on Equine Influenza

Equine influenza (EI) is a major respiratory disease of equids caused by type A influenza viruses (equine influenza virus: EIV). In naïve and unprotected animals, clinical signs of disease typically occur 48 h or more after infection with EIV and are characterised by an elevation of body temperature, nasal discharge, cough, and sometimes respiratory distress. EIV is highly contagious and released in large quantities during coughing episodes [1], which allow rapid spreading of the disease by inhalation of infectious aerosols [2,3]. Equine influenza is associated with high morbidity; mortality is rare but occurs occasionally in foals and donkeys [1,4,5,6]. In adults, mortality is generally a consequence of poor health condition and/or secondary bacterial infection. 

**Figure 1:**
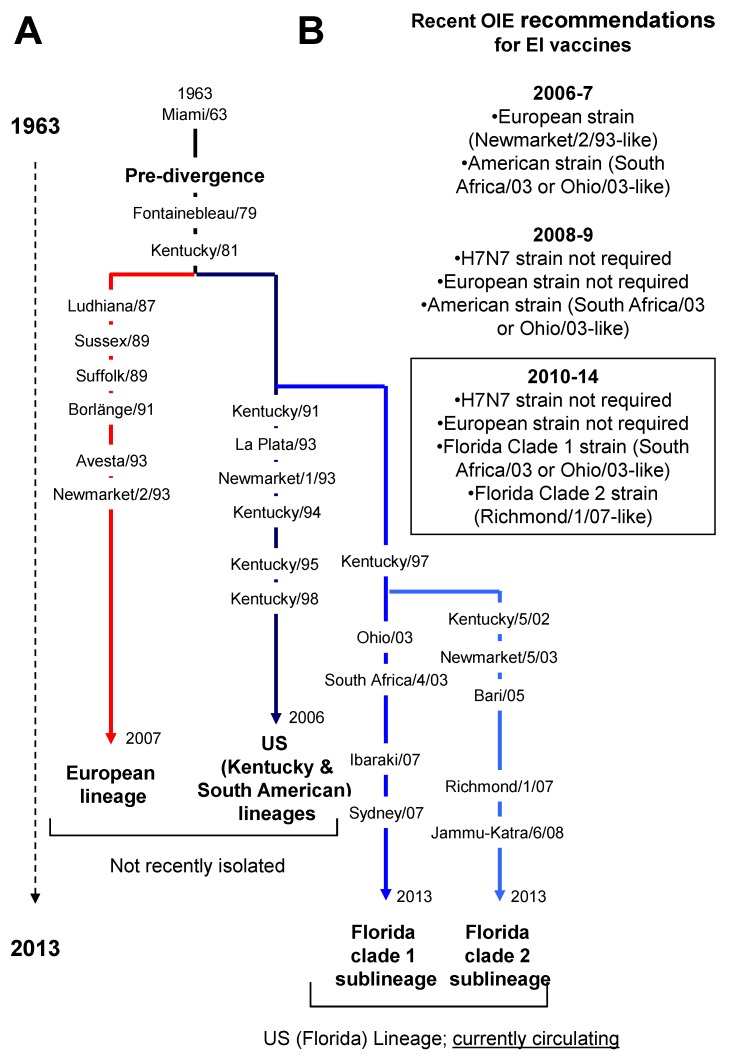
(**A**) Schematic of equine influenza virus (EIV) evolution. All main sub-lineages and EIV strains reported in this review are indicated; (**B**) Recent World Organisation for Animal Health (OIE) recommendations from the OIE expert surveillance panel on EI vaccine composition.

In horses, EIV isolates are classified by their subtype and named on the basis of location and year of isolation. Two different subtypes have been designated based on antigenic properties of the haemagglutinin (HA) and neuraminidase (NA) envelope glycoproteins. The H7N7 subtype (A/equine/1/Prague/56 as prototype strain) was first identified in Eastern Europe in 1956 [7], but has not been isolated from horses for over 20 years [8,9] and is therefore presumed extinct from horses. Equine influenza H3N8 viruses (A/equine/2/Miami/63 as prototype strain) were first isolated in North America in 1963 [10] and are still circulating [11]. EIV HA molecules undergo natural mutation known as antigenic drift, which confers the virus ability to modify its host receptor cell binding specificity and to evade host immunity. Such a mechanism drives the evolution and natural selection of EIV, and phylogenetic analyses have identified a divergence of the H3N8 subtype in the late 1980s, giving rise to the Eurasian and American lineages [12] (Figure 1). The origin of an evolution bottleneck at the end of 1980s is currently argued and could be due to an increased use of EI vaccines [13], as opposed to a large epidemic (discussed at the 2nd International Symposium on Neglected Influenza Viruses, 7th–8th March 2013, Dublin, Ireland). The American lineage has further diverged into the South American, the Kentucky and the Florida sublineages [14], the latter of which has since divided into two separate clades, designated clade 1 and 2 [15,16]. All of the recently isolated viruses in North America and Europe belong to the Florida clades 1 and 2 sublineages, respectively [16,17]. The evolution of EIV has been the subject of recent review [18,19,20,21]. 

### 1.2. Equine Influenza Outbreaks in the Recent Years

Despite the development and commercialisation of EI vaccines for almost 5 decades, H3N8 EIV is still circulating and considered endemic in Europe and North America [16,17,22,23]. Several major EI outbreaks were reported in the last seven years. In 2007, 200 to 300 incompletely or unvaccinated horses were affected in Sweden [24]. The same year, around 1500 horses were affected by EI in Japan. Several cases were reported in the facilities of the Japan Racing Association (JRA) despite mandatory and regular EI immunisation [25,26]. This outbreak was associated with use of an outdated whole inactivated EIV vaccine [25]. Vaccine protection against A/eq/Ibaraki/07 (representative strain of the Japanese outbreak [16,26]) was partial, but contributed to reduce morbidity rate, with around 19% of the vaccinated population showing a subclinical form of the disease [25,26]. Australia had remained free from EI prior to 2007, when over 75,000 horses were infected with EIV. The representative virus A/eq/Sydney/2888-8/07 was classified as a member of the Florida clade 1 sublineage, typical of viruses recently isolated in North America and closely related to A/eq/Ibaraki/07. This outbreak may have been facilitated by the importation of a subclinically infected horse, which had responded poorly to recent vaccination [27,28,29], the breach of quarantine, subsequent virus escape, and the absence of a vaccination programme in Australia. Similar scale outbreaks occurred in Mongolia, Northern China and India from 2007 to 2011 [30,31]. During these epidemics thousands of equids were affected, including domestic, wild and competition horses and donkeys, the majority of which were presumably unvaccinated [32]. The Chinese and Indian EIV outbreak strains were related to the Florida clade 2 sublineage [33,34]. Large outbreaks were reported in South America in 2012, associated with EIV strains of the Florida clade 1 sublineage [35]. Several outbreaks of limited size were reported in Europe in recent years (e.g., LaBaule outbreak in 2012, and numerous cases in UK in 2013, [36,37]). Based on their immunological status, a study in Ireland has recently indicated that weanlings, teasers and non-thouroughbreds horses were probably the most susceptible to infection [38].

### 1.3. Protection against Equine influenza 

**Figure 2:**
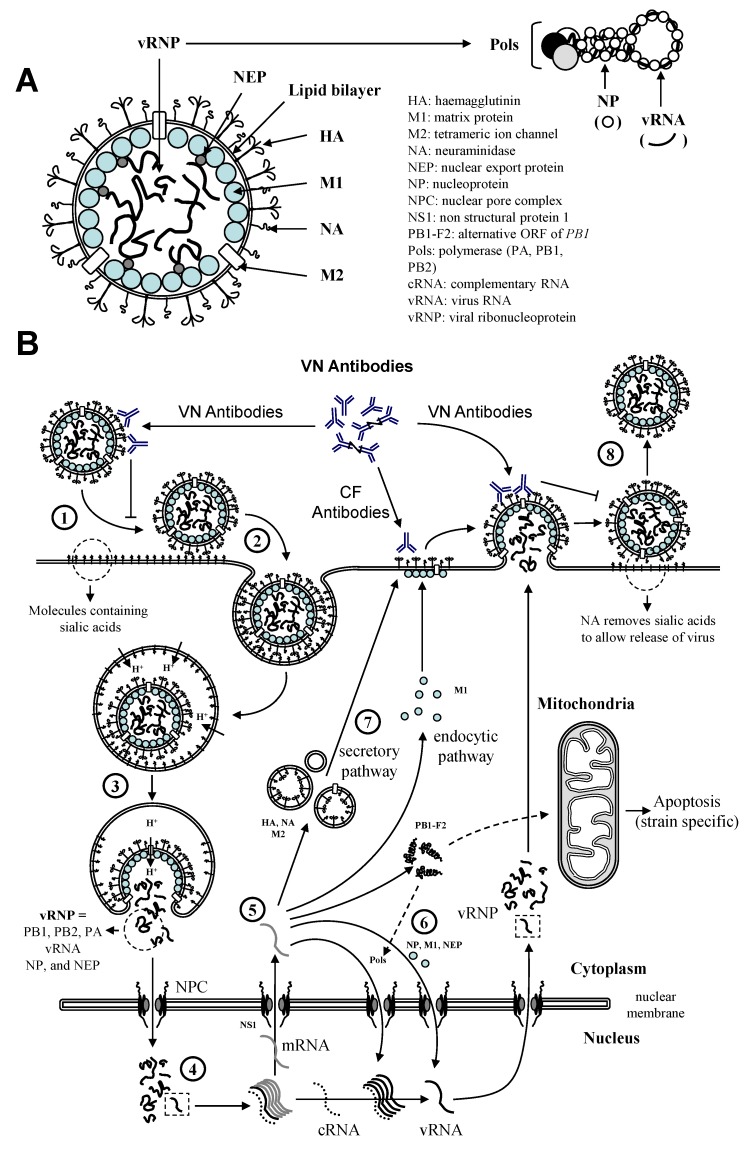
(**A**) EIV structure; (**B**) The reproductive cycle of influenza A virus. The virus binds to receptors on the surface of the cells (1) and is internalized into endosomes (2). Modification of pH in both the endosome and the virus induces fusion and uncoating of the virus (3). vRNP are released into the cytoplasm and imported into the nucleus where their replication takes place (4). mRNA are produced and exported into the cytoplasm for protein synthesis (5). This is controlled by NS1. These viral proteins will either assist replication of the virus and formation of vRNP into the nucleus (6), or form new viruses at the cell surface (7). Progeny viruses are assembled and bud from the cell membrane (8). CF = complement fixing; VN = virus neutralising.

Natural or experimental infection with EIV induces protection against re-infection with a homologous or closely related strain for several months [39,40] (reference 40: study sponsored by Horserace Betting Leavy Board), depending on the individual. The level of EIV-specific antibodies, measured by single radial haemolysis (SRH) or haemagglutination inhibition (HI) assays, is a correlate of protection against homologous EIV strains. Reduced clinical signs of disease and resistance to infection with an EIV strain homologous to the vaccine strain were observed in animals with SRH antibody levels of >85 mm² and >120–154 mm², respectively [41,42,43,44]. Current field data still support this correlation [45]. Antibodies specific to influenza virus HA and NA molecules act by neutralising the virus prior to infection of the respiratory epithelium and by inhibiting virus release after replication in infected cells, respectively [1,18] (Figure 2). Virus neutralising (VN) antibodies are essential to limit the spread of the disease [46,47]. Complement fixing (CF) antibodies target EIV infected cells in order to clear the infection. Both HA and NA are important influenza vaccine components. Immunity to EIV HA has been well described. In contrast, NA immunity is poorly characterised in the horse. Vaccination against EIV, which is an efficient method of prevention, relies on the antigenic homogeneity between the vaccine and circulating EIV strains. Therefore, a constant monitoring of the antigenicity of circulating EIV strains is essential to select appropriate vaccine strains and to ensure that vaccines remain up to date. The OIE (World Organisation for Animal Health) expert surveillance panel on EI vaccine annually reviews laboratory and epidemiological data about worldwide EIV circulation. EIV gene sequences (primarily HA and NA) and antigenic variation assessed with predictive tools and models (e.g., immune-reactivity using strain specific ferret sera) and quantified by cartography are analysed in order to anticipate the impact of EIV antigenic drift on vaccine protection, and to deliver an annual recommendation on vaccine strain composition. Since 2011, the recommendation approves the incorporation of both Florida clade 1 and clade 2 EIV representative strains of the Florida sublineage in vaccine [48]. The inclusion of H7N7 virus and H3N8 EIV of the European lineage is no longer supported. These recommendations remained unchanged [11]. To date, one EI vaccine has been fully updated to meet to the current recommendation.

Protection against EI has also been reported in the absence of antibodies at the time of re-infection [49]. Significant protection against the Australian outbreak strain A/eq/Sydney/2888-8/07 was observed in four ponies exposed 18 months earlier to the phylogenetically-related isolate A/eq/South Africa/4/03. No or low levels of circulating antibodies were measured at the time of re-infection [40]. In these conditions, protection is likely to involve stimulation of cell-mediated immunity (CMI) to clear infected cells, to reduce the severity of infection, to limit morbidity and improve recovery. Stimulation of macrophages, natural killer cells, cytotoxic T lymphocytes (CTL) directly targeting EIV infected cells, and/or armed EIV-specific T lymphocytes necessary for a rapid mobilisation of memory B cells and subsequent antibody synthesis is essential. As for other influenza A viruses, CMI stimulation could be considered as a co-correlate of protection against EIV [50]. The main CTL EIV-antigen targets have not been identified but are believed to be conserved internal viral proteins (like for other influenza viruses), which may be important for cross-protection [51].

## 2. Systematic Review: Methods

This review is written following the PRISMA statement [52]. The protocol, which is described here, was not registered with PRISMA.

### 2.1. Search Strategy (Figure 3)

In December 2013, a systematic literature search was conducted on equine influenza vaccination in horses for prevention/treatment of equine influenza. Electronic databases were searched using MEDLINE (PubMed with date limitation from 2006 to present) and Scopus (2006 to present). The search terms specified were “equine influenza vaccine OR equine influenza vaccination”. Conference proceedings, manufacturing reports, patent and round-table discussions were also considered. The language of publication is mostly English, but not exclusively. Publication date ranged from 2006 to the present.

**Figure 3:**
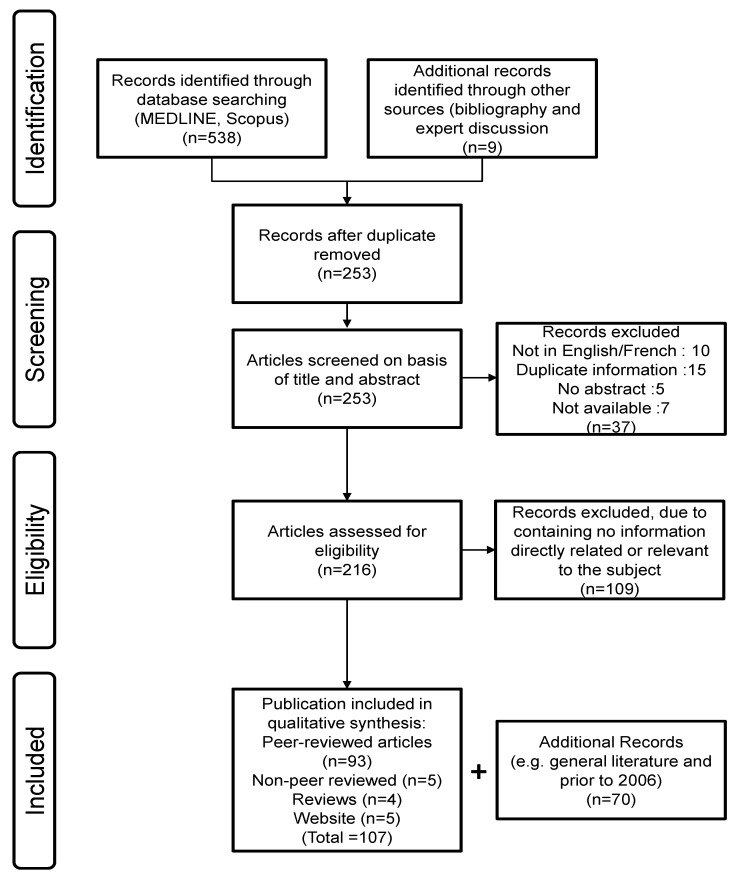
Systematic review process.

### 2.2. Publication Selection

The publications were screened on title and abstracts. Those describing the field and/or experimental use of equine influenza vaccines and/or their characterisation were included. Publication date restriction was imposed (2006 to present) but no publication status restriction was imposed. Publications not in the English or French language were considered provided an abstract was available. Records were rejected if they were duplicates of data available as published articles. Studies published both in abstract and full paper forms were only evaluated in their full paper format.

### 2.3. Data Collection Process and Items

Each selected publication was entered into a reference manager system (Endnote X2). The selected publications were ordered by primary authors to avoid duplication. Information was extracted from each included study and reports on type of EI vaccine (e.g., whole inactivated virus vaccine), context of the study (e.g., experimental clinical trial or field study, outbreak and/or vaccine breakdown reports), type of intervention (dose, duration and frequency), number of participants in each group and type of outcome (e.g., humoral response and/or cell-mediated immunity).

### 2.4. Risk of Bias in Individual Studies

Conference proceedings, manufacturing reports, round-table discussions and abstracts were considered. By their nature and the limited amount of data available, results and/or opinions reported in these publications may be biased. The term “significant” was used when the statistical significance of results presented was reported in the considered report. Terms such as “anecdotal, anecdotally or expert opinion” were used to qualify data extracted from conference proceedings, expert opinion and/or round-table discussion. When known, study sponsors were indicated in text in case of potential conflict of interest.

## 3. Equine Influenza Vaccination

**Figure 4:**
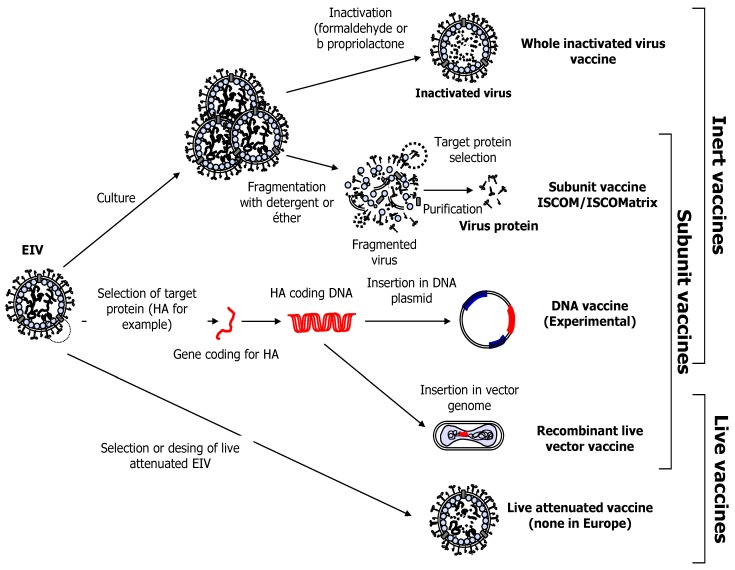
Different types of EI vaccines.

Vaccination is one of the most effective tools, alongside isolation, movement restriction and basic biosecurity measures [53,54], to prevent EIV infection or to limit its consequences. Equine influenza vaccines have been available since the 1960s and vaccination is mandatory in the UK for racing Thoroughbreds since 1981. Today, EI vaccines commercialised worldwide could be differentiated into three groups based on their technology (*i.e.*, whole inactivated/sub-unit ISCOM-Matrix or ISCOM, live-attenuated and viral-vector based; Table 1, Figure 4). Several EI vaccines are licensed and sold in Europe [19]. These vaccine technologies have been previously described [55]. 

The following elements are taking into account for the commercialisation of an EI vaccine:

- The safety of the product
- A demonstrated efficacy against at least one of the strains contained in the vaccine [56,57]. The serological response could be used to confirm efficacy if the protection induced by a vaccine strain has previously been demonstrated by experimental challenge infection.
- Protection: a significant reduction in clinical signs of disease (only slight signs recorded in vaccinated animals) and virus shedding is expected, when compared with unvaccinated controls animals. Neutralising immunity (*i.e.*, neutralisation of virus leading to an absence of infection and subsequent seroconvertion) is rare.

The licensing requirement have been recently summarised by Woodland [57].

The conventional EI vaccination schedule of naïve horses (except for live attenuated EI vaccine) consists of a primary course of two immunisations, (4 to 6 weeks apart according to vaccine labels, 21 to 92 days according to Racing authorities), followed by a third (boost) immunisation (5 to 6 months later according to product labels, 150 to 215 days according to racing rules) and subsequent bi-annual or annual immunisation (Federation Equestre Internationale, UK Jockey Club rules for equine influenza vaccination; [58]).

**Table 1:** Examples of commercial EI vaccines, classed by technologies. Several of these vaccines are commercialised as EIV vaccine alone or in combination with Tetanus (such combination will be reflected in the name of the vaccine, e.g., Duxaxyn IE-T Plus).

Technology	Example of Vaccine	Company	Adjuvant	Antigens	EIV Strains
Whole inactivated Sub-unit I SCOM/ISCOM-Matrix	Duvaxyn^Tm^ IE Plus	Elanco	Carbopol	whole virus	Newmarket/1/93 (H3N8) Suffolk/89 (H3N8)Prague/56 (H7N7)
	Calvenza^®^-03 EIV	Boehringer Ingelheim Animal Health	Carbopol	whole virus	Newmarket/2/93 (H3N8)Kentucky/2/95 (H3N8)Oiho/03 (H3N8)
	Equilis Prequenza (updated 2013)	MSD Animal Health	ISCOM-Matrix	whole virus	Newmarket/2/93 (H3N8)South Africa/4/03 (H3N8)
	Equilis Prequenza	MSD Animal Health	ISCOM-Matrix	Sub-unitHA	Prague/56 (H7N7)Newmarket/1/93 (H3N8)Newmarket/2/93 (H3N8)
	Equip^TM^ F	Pfizer Ltd.	Self adjuvanting (ISCOM)	Sub-unit mainly HA and NA	Newmarket/77 (H7N7)Borlänge/91 (H3N8)Kentucky/98 (H3N8)
Modified live EIV	Flu Avert^®^ I.N.	Intervet/Schering-Plough Animal Health (US)	na	whole virus	Attenuated, cold adapted EIV: Kentucky/91 (H3N8)
Viral-vector based	PROTEQ FLU™	Merial Animal Health Ltd.	Carbomer	HA	Ohio/03 (H3N8)Newmarket/2/93 (H3N8)
	PROTEQ FLU™(updated 2014)	Merial Animal Health Ltd.	Carbomer	HA	Ohio/03 (H3N8)Richmond/1/07 (H3N8)

Na = not applicable; HA = haemagglutinin; NA = neuraminidase.

### 3.1. Vaccine Strain Mismatch and Recent EI Outbreaks

It is widely accepted that a close antigenic relationship between an EI vaccine and field circulating EIV strains is essential to protection. This is illustrated by several examples of vaccine breakdown in the last decade. In 2004, an EI outbreak was reported in Croatia. The EI vaccine used at the time contains A/eq/Miami/63 and A/eq/Fontainebleau/79. Taking into account the extended mismatch between the vaccine and outbreak EIV strains, the absence of protection is not surprising [59]. In 2003, a significant EI outbreak was reported in Newmarket (UK). Most of the infected horses had been recently vaccinated with representative EIV strains of both European and American lineages (A/eq/Newmarket/2/93 and A/eq/Newmarket/1/93, respectively) [60,61]. The outbreak EIV isolates (A/eq/Newmarket/5/03 as representative) were related to EIV strains of the Florida clade 2 sub-lineage. Nine consistent amino acid substitutions were identified in the consensus HA1 sequence of the outbreak strains, when compared with A/eq/Newmarket/1/93 (one mutation was a likely consequence of adaptation after amplification in embryonated eggs) [60]. South Africa was also affected in 2003. However, the outbreak strains (A/eq/South Africa/4/03 as representative) belonged to the Florida clade 1 sub-lineage. These outbreaks associated with clear vaccine breakdowns prompted a strain update recommendation from the OIE expert surveillance panel on EI vaccines. In 2005, 24 out of 33 horses recently vaccinated (<3 months) with a whole inactivated EIV vaccine adjuvanted with carbopol/aluminum hydroxide and containing the strain A/eq/Newmarket/1/93 were infected with EIV in Italy. The outbreak strain A/eq/Bari/2005, was closely related to the A/eq/Kentucky/5/02 strain (clade 2) [16,62,63], which was introduced in Italy after the 2003 outbreak in the UK [60,61]. Sequence analysis of the outbreak isolate A/eq/Bari/2005 revealed four amino acid substitutions in the HA1 subunit when compared with the vaccine strain HA1, which may have been responsible for the reduced vaccine protection [63]. Only four amino acid changes located in two separate antigenic sites represent a significant antigenic drift for human influenza A viruses [64]. Similar rules may apply to EIV [65]. The Japanese outbreak in 2007 was associated with use of an outdated whole inactivated EIV vaccine containing the strains A/eq/La Plata/93 (South American lineage) and A/eq/Avesta/93 (European lineage) [25,66]. There were 11 amino acid differences between the HA1 molecules from the vaccine strain A/eq/La Plata/93 and the representative outbreak strain A/eq/Ibaraki/07. HI antibody response induced by immunisation with A/eq/La Plata/93 has been shown to cross-react with more recent EIV strains such as A/eq/Ibaraki/07 or A/eq/Richmond/1/07 [67]. However, the HI antibody titres induced by the A/eq/La Plata/93 vaccine were <60 when tested 6 months after the last immunisation [66] (study sponsored by Japan Racing Association). Such HI titres were estimated to be insufficient to provide complete clinical protection (threshold = HI titres >80) [67], which was supported with epidemiological data recorded in Japan in 2007. The inactivated EIV vaccine currently commercialised in Japan was recently updated with the incorporation of the EIV A/eq/Ibaraki/07 strain (Florida clade 1). The antibody response induced by this vaccine was tested against A/eq/Richmond/07 (Florida clade 2) shortly after the 2010 OIE recommendation to include a representative strain of the Florida clade 2 lineage in EIV vaccine [67]. Results of this study indicated that the HI antibody titres tested against the EIV strains A/eq/La Plata/93 and A/eq/Richmond/07 were similar after immunisation with the Japanese updated inactivated EIV vaccine. However, the level of cross-protection induced by this vaccine against clade 2 EIV strains remains unknown. In 2008–2009, an outdated inactivated EI vaccine containing the strain A/eq/Ludhiana/87 was available in India but not routinely used [30]. A/eq/Ludhiana/87 belongs to the European H3N8 lineage, closely related to A/eq/Suffolk/89 [34]. The representative Indian outbreak isolate A/eq/Jammu-Katra/6/08 is antigenically related to the Florida clade 2 sublineage of the American H3N8 lineage. Antibody cross-reaction between European and American H3N8 lineage is limited [68] and heterologous immunisation has shown lower virological protection [69,70]. In this context, the impact of the A/eq/Ludhiana/87 vaccine would have been limited during the 2007 Indian EI outbreak.

These outbreaks highlight the importance of antigenic homogeneity between the vaccine and circulating EIV strains to induce and maintain a long lasting protection. Significant levels of protection against distantly related EIV strains or strains of mild pathogenicity may be observed shortly after immunisation with an out-dated EI inactivated vaccine [71] (study sponsored by Fort Dodge Animal Health). A whole inactivated EI vaccine containing the EIV strain A/eq/Newmarket/1/93 was shown to protect against the outbreak isolates A/eq/South Africa/4/03 (H3N8; Florida clade I) [72] (study sponsored by Fort Dodge Animal Health), A/eq/Sydney/2888-8/07 [71] and A/eq/Richmond/1/07 [73]. Vaccinated ponies were significantly protected against these outbreak isolates when experimentally infected at the peak of immunity, 2 weeks after the second immunisation. Overall virus shedding was also reduced in immunised animals [71,72,73]. However, the quality of mid- and long-term cross-protection is often unknown and likely to be inferior to protection induced by an equivalent EI vaccine matching the circulating isolates. The HA1 molecule from the EIV vaccine strain A/eq/Newmarket/1/93 possesses 17 amino acid differences when compared with the HA1 molecule of A/eq/Sydney/07. Eight amino acid differences are located in the antigenic sites identified in H3 influenza viruses [26]. The *P_epitope_* value is a sequence-based antigenic distance measure between two EIV strains (*i.e.*, fraction of amino acid substitution in the dominant HA epitope). The correlation between *P_epitope_* value and vaccine effectiveness was demonstrated for some subtype of human A influenza viruses. A negative correlation between the EIV *P_epitope_* and EI vaccine strain efficacy was recently reported [74]. The *P_epitope_* correlation reported was based on efficacy studies using crude whole inactivated EIV vaccines. The statistical significance of this negative correlation was dependent of the studies included for the calculation. Further work would be required to refine the *P_epitope_* calculation for EIV and to evaluate its potential as providing complementary information for the EI vaccine strain selection. However, it is important to note that EI vaccine used in this study were of relatively simple composition, like human influenza A vaccines, in contrast to modern EI vaccine technologies that not only differ in terms of antigen load and nature, but also contain powerful adjuvants and stimulate CMI. 

### 3.2. Whole Inactivated and Sub-Unit EIV Vaccines

Whole inactivated EIV vaccines were the first type of vaccine to be developed and were the predominant type of EI vaccine available for decades. Equine influenza viruses are grown in embryonated hens’ eggs or cell culture [75,76] prior to chemical inactivation.

#### 3.2.1. Whole Inactivated EI Vaccine and Immune Response

The protection induced by first generations of whole inactivated, aluminium hydroxyde adjuvanted, EI vaccine primarily relied on stimulation of high antibody levels. Aluminium hydroxyde is known to drive a Th2, antibody orientated immune response [77]. The use of new adjuvants in later version of this type of vaccine may have changed the nature of the protective immunity induced. Whole inactivated EI vaccines not only target antigenically variable EIV surface antigens (*i.e.*, HA and/or NA) but also stimulate immune response to more conserved EIV proteins (such as NP or matrix), which are believed to induce some level of cross protection [78]. However, such protection remains to be demonstrated in the horse. Evidences of CMI stimulation have been recently reported [73,79] (reference 73: study sponsored by Elanco Animal Health; reference 79: study sponsored by the Irish Department of Agriculture). The pro-inflammatory cytokines IFNalpha and IL-6, which may correlate with virus shedding and clinical signs of disease [80], were significantly decreased in vaccinated animals experimentally infected with EIV [81] (study sponsored by the Irish Department of Agriculture). Two recent studies have also shown that a whole inactivated EIV vaccine, carbopol adjuvanted, stimulate higher levels of SRH antibody when compared with other commercialised EI vaccines [79,82] (studies sponsored by the Irish Department of Agriculture). A similar observation was previously made with different carbopol adjuvanted whole inactivated EI vaccines [83] (study sponsored by Intervet Inc.). This increased antibody response has not yet been fully explained. The use of a B-class CPG ODN 2007 adjuvant in combination with a commercial killed EIV vaccine significantly increased the stimulation of SRH antibody in horses when compared with the vaccine alone. However, this adjuvant did not significantly improve the vaccine protection when challenged with A/eq/Kentucky/99 [84].

Overall, stimulation of CMI and higher levels of antibody may balance in some measure the current mismatch between vaccine and circulating EIV strains. High levels of cross-reactive antibody may provide significant protection in the context of an imminent EI outbreak, when high herd immunity needs to be rapidly induced and updated vaccines are not yet available. However, the duration of such a protection may be reduced and remains to be assessed. A new whole inactivated EIV vaccine adjuvanted with ISCOM-Matrix and containing the A/eq/South Africa/4/03 strain (2004 OIE recommendation for vaccine composition) is now commercially available in some European countries. Clinical and virological protection was reported by the manufacturer when challenged at the onset of immunity with the strain A/eq/Richmond/1/07 [85]. 

#### 3.2.2. Sub-Unit EI Vaccine, ISCOM/ISCOM-Matrix Adjuvanted

**Figure 5:**
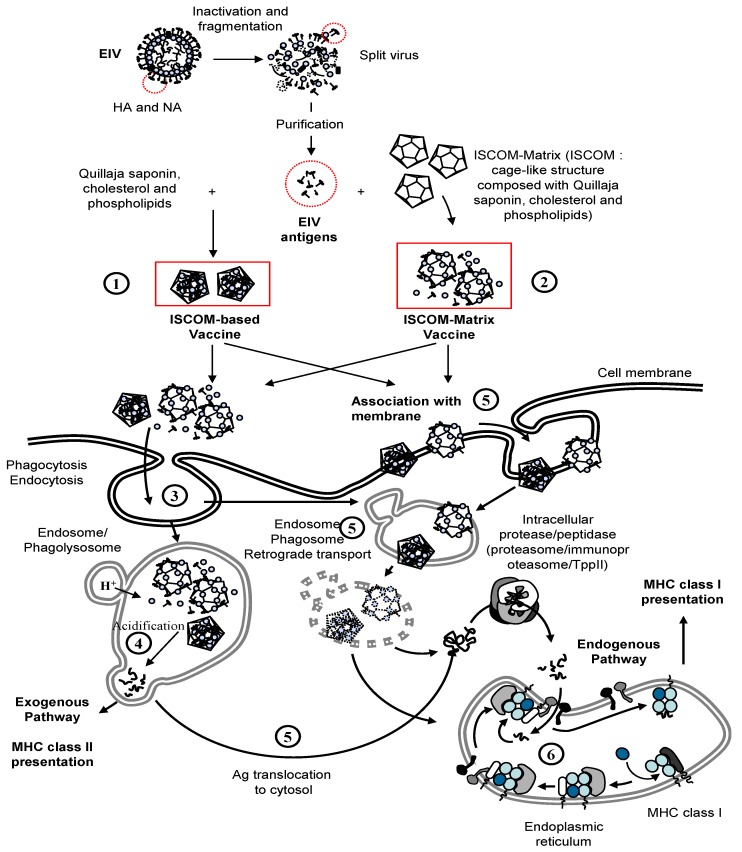
Immuno-Stimulating Complex adjuvanted vaccines (ISCOM) and ISCOM-Matrix vaccine, uptake and major histocompatibility complex (MHC) presentation. Purified EIV antigens (e.g., HA and NA) are directly mixed with Quillaja saponin, cholesterol and phospholipids to produce ISCOM-based vaccine (1) or mixed with preformed ISCOM to produce ISCOM-Matrix-based vaccine (2). Exogenous pathway and antigen presentation by MHC class II molecules. The antigen/ISCOM/ISCOM-Matrix complex is engulfed in phagosomes (3) and enter the exogenous pathwary of antigen presentation after degradation by proteases into short peptides in acidified endosomes (4). The MHC-class II-peptide complex is recognised by the T cell receptor (TCR) of antigen-specific CD4^+^ T lymphocytes. Cross-presentation of antigen by MHC class I molecules. The antigen/ISCOM/ISCOM-Matrix complex could enter the cell through interaction with the cell membrane, phagocytosis, endocytosis, or similar process (5). The antigens are translocated to the cytosol and reach the endogenous pathway of antigen presentation (6). The complex MHC class I-b2m-peptide is recognised by the T cell receptor (TCR) of an antigen-specific CD8^+^ T lymphocyte.

The immuno-stimulating complex (ISCOM) technology is based on the particulate ISCOM-Matrix adjuvant, which is composed of phospholipids, cholesterol and Quil-A (Quillaja) saponin (matrix component) [86,87]. As shown in Figure 5, the ISCOM technology regroups the ISCOM-based and ISCOM-Matrix adjuvanted vaccines, which differ in terms of formulation. ISCOM-based vaccines contain hydrophobic antigen and/or membrane proteins that are directly mixed with the matrix component to give stable, self-adjuvanting particles, held together by hydrophobic interactions [88]. ISCOM-Matrix-based vaccine corresponds to the association of already prepared matrix particles with purified antigens. The antigen presentation is believed to be similar after immunisation with ISCOM-based or ISCOM-Matrix vaccines [87,89,90]. Exogenous antigens adjuvanted with ISCOM/ISCOM-Matrix are processed by both endogenous and exogenous pathways and presented via major histocompatibility complex (MHC) class I and II molecules, respectively [91,92]. The exogenous pathway involves phagocytosis of the antigen/ISCOM/ISCOM-Matrix complex by antigen presenting cells (APC). However, the complex is also thought to interact with the cell membrane and subsequently with intracellular lipid membranes [86,90]. ISCOM/ISCOM-Matrix structures have been localised within the cytoplasm and vesicular compartments in APC [89,90,93]. The membrane-disrupting properties of the Quil-A saponin is believed to play a role in the antigen escape from endosomes/lysosomes and release into the cytoplasm to enter the endogenous pathway of antigen presentation [86]. The ISCOM/ISCOM-Matrix adjuvant is also a powerful immunomodulator that induces the synthesis of pro-inflammatory, Th1 and Th2 cytokines [86,88,94] and up-regulates expression of MHC and co-stimulatory molecules in APC [91,95]. These activities remains to be demonstrated in horses. Markers of an acute phase response, with elevated serum amyloid level, were reported after immunisation with the ISCOM-based EI vaccine [96].

In terms of protection, the ISCOM-based vaccine containing the strains A/eq/Newmarket/77 (H7N7), A/eq/Kentucky/98 (American lineage, [H3N8]) and A/eq/Borlänge/91 (Eurasian lineage, [H3N8]) was shown to significantly reduce both clinical signs of disease and virus shedding after experimental infection with the EI outbreak isolates A/eq/South Africa/4/03 [97] (study sponsored by Schering Plough Animal Health), A/eq/Sydney/2888-8/07 [98] (study sponsored by the Horserace Betting Levy Board) or anecdotally against A/eq/Richmond/1/07 when challenged at the peak of immunity (*i.e.*, two weeks post second vaccination = V2) [97]). Similar protection has been anecdotally reported in ponies vaccinated twice with the ISCOM-Matrix-based EI vaccine, which contains purified HA and NA subunits from EIV strain A/eq/Prague/1/56 (H7N7), A/eq/Newmarket/1/93 (H3N8, American lineage) and A/eq/Newmarket/2/93 (H3N8, European lineage), against the EIV outbreaks strains A/eq/South Africa/4/2003, A/eq/Ohio/2003, and A/eq/Newmarket/5/2003 [99,100] (Studies sponsored by Intervet Int.). When used in the face of an EI outbreak in Ireland, boost immunisation with the ISCOM-Matrix EI vaccine prevented the appearance of new cases in several premises [23]. 

Protection induced by ISCOM/ISCOM-Matrix-based EI vaccines is based on a well characterised antibody response [101,102,103,104] (reference 101: study sponsored by Intervet/Schering Plough Animal Health; references 102–104: studies sponsored by Schering Plough Animal Health). Stimulation of CMI after vaccination with ISCOM technology adjuvanted influenza vaccine has been described in humans [50] but has only recently been demonstrated in the horse. Stimulation of EIV-specific IFNgamma synthesis has been measured in ponies immunized with an ISCOM-Matrix [79,105] (study sponsored by Intervet/Schering Plough Animal Health) or an ISCOM-based adjuvanted EIV vaccine [97]. 

Anecdotal data reports that the EIV-specific T lymphocyte response was long lasting and measurable up to one year after boost vaccination with the ISCOM-based EIV vaccine. Levels measured at this time point were similar to those measured in ponies one year after experimental infection with A/eq/South Africa/4/03. Clinical and virological protections against the EIV strain A/eq/Richmond/1/07 were also measured 12 weeks after the second immunisation with an ISCOM-based EI vaccine. Vaccinated ponies were significantly protected but still able to transmit EIV to naïve sentinel ponies for up to 8 days post experimental infection [106] (study sponsored by Racing Victoria Ltd.). This result illustrates the difficulty encountered by EI vaccine to protect during the immunity gap, a period of susceptibility to EIV infection observed in the weeks preceding the third immunisation. The immunity gap will be discussed later in this review. The long-term protection induced by the ISCOM-Matrix-based EI vaccine was confirmed when immunised horses were experimentally infected with A/eq/Kentucky/8/95, 54 weeks after the third immunisation [107] (study sponsored by Intervet/Schering Plough Animal Health).

### 3.3. DNA Vaccination against EIV

DNA vaccination corresponds to the injection of DNA plasmids expressing genes from specific pathogens, encoding whole antigenic proteins, or simply epitopes of these proteins. Antigens are directly expressed in the target cells after immunisation. In the horse, skin and mucosal immunisation with EIV DNA vaccines induced significant levels of protection against experimental infection with EIV. However, the multiple site and frequency of administration (up to 60 sites of injection per immunisation in one study) were obstacles for DNA vaccine development and use in veterinary practice [108,109,110]. Intralymphatic immunotherapy (ILIT) has been recently tested as a new vaccination approach in the horse. Five influenza negative mixed-breed ponies received one injection of a plasmid containing the EIV HA gene from A/eq/Kentucky/1/81 in the submandibular lymph node on days 0, 28 and 98. All immunised ponies developed an EIV-specific antibody response after the second and third DNA immunisation. The amplitude of this response was similar to the response measured after experimental infection with A/eq/Kentucky/91 but significantly lower than the response detected in animals conventionally vaccinated with the canarypox-based EIV vaccine [111]. The intranodal DNA immunisation is a positive development in equine vaccination and therapy as it allows the delivery of vaccine directly at the site where B and T lymphocytes are primed, which should improve vaccine immunogenicity. However, the field feasibility of this type of immunisation will have to be evaluated, alongside the risk of decreased efficacy if the vaccine dose is not accurately or entirely injected in the submandibular lymph node. More recently, DNA vaccine expressing the HA molecule from A/eq/Ohio/03, A/eq/Bari/05 and A/eq/Aboyne/05 were tested in ponies. Ponies were experimentally infected with the EIV strain A/eq/Ohio/03, 8 weeks after the last immunisation (three immunisations, 4 weeks apart). Results presented in this study revealed that needle free intra-dermal administration of the DNA vaccine was as efficient, in terms of immunogenicity and protection, than conventional intra-muscular administration of the same vaccine. Multivalent DNA immunisation induced significant level of clinical and virological protection after challenge infection, when compared to unvaccinated horses [112]. These results are promising for the field feasibility of DNA vaccine.

### 3.4. Modified Live EIV Vaccine

Modified live EIV vaccine contains a live attenuated influenza virus that retains its ability to infect the host and their immunogenicity. This type of vaccine stimulates a long lasting immune response, involving both antibodies and cell-mediated immunity, which could theoretically improve cross-protection. The attenuated influenza virus should have limited replication ability, inducing no or limited clinical signs of disease and should not be excreted after immunisation to avoid transmission to other individuals. A reversion of the vaccine strain, a possible recombination with a circulating field influenza virus in infected patients, and the induction of disease in immune-compromised individuals have always been potential risks associated with the use of live attenuated influenza vaccines.

A cold adapted and temperature sensitive live equine influenza vaccine (a live virus that does not replicate at the higher temperatures found in the lower respiratory tract and lungs) is currently commercialised in the US (Flu Avert^®^ I.N. Vaccine). This live vaccine is intra-nasally administered and is derived from the wild-type A/eq/Kentucky/1/91 (H3N8) EIV strains. The live attenuated EIV vaccine was successfully tested for efficiency against EIV, stability, low transmission to unvaccinated horses, and the absence of side effect in immune-depressed animals [113,114]. To our knowledge, no side effects have been reported since the commercialisation of the EIV live attenuated vaccine.

Reverse genetics allows the generation of artificial recombinant influenza viruses using cloned DNA plasmids [115] (described and illustrated in [18]). Three recombinant EIV with a carboxy-terminal truncation in their NS1 genes have been engineered by reverse genetics [116]. Recombinant equine influenza viruses were recovered after transfection of MDCK (Madin Darby Canine Kidney) cells with DNA plasmids coding for each of the eight influenza RNAs (HA, M, NA, NS, PA, PB1 and PB2) simultaneously with plasmids expressing the proteins PA, PB1, PB2, NP and NS1 [117]. Truncation in the NS1 gene prevents virus replication in interferon competent cells or *in vivo*. Horses were inoculated twice 4 weeks apart with the recombinant NS1 truncated EIV. They did not develop clinical signs of disease after immunisation but shed detectable levels of infectious vaccine virus for several days. All vaccinated horses had seroconverted to EIV by V2 and were experimentally infected with A/eq/Kentucky/5/02, 4 weeks later. Both clinical signs of disease and virus shedding were significantly reduced in vaccinated horses when compared with naïve horses [118]. These results support the promising development of future EI vaccines by reverse genetics, which provide the possibility to rapidly and easily modify the antigenic characteristics of the vaccine strain by genetic manipulation.

### 3.5. Non-Influenza Viruses as Vaccine Vector

#### 3.5.1. Canarypox-Based Vaccine

Recombinant poxviruses are infectious particles containing segments of foreign DNA that are expressed as antigens after immunisation and *in vivo* cell infection (Figure 4). The avian poxviruses are usually safe vaccine vectors in mammals because they undergo an incomplete replication cycle in mammalian cells. The canarypox-based vaccine expressing HA molecules from A/eq/Kentucky/94 and A/eq/Newmarket/2/93 (American and Eurasian lineages, respectively) was shown to significantly reduce clinical signs and virus shedding in ponies experimentally infected with A/eq/Newmarket/5/03 [119,120] (studies sponsored by Merial Ltd.) or A/eq/Sydney/2888-8/07 [98] (study sponsored by the Horserace Betting Levy Board), 2 weeks after the second vaccination. A/eq/Newmarket/5/03 is a member of the Florida sublineage clade 2 viruses (commonly circulating in the UK) and was responsible for the large outbreak seen in vaccinated horses in Newmarket in 2003 [60]. This vaccine has been updated twice. It contains the EIV strain A/eq/Ohio/03, as recommended by the OIE in 2006 [121], to replace A/eq/Kentucky/94 as a representative of the American sublineage. The A/eq/Newmarket/2/93 strain has also recently been replaced with A/eq/Richmond/1/07 in order to meet the last OIE recommendation [11]. 

The onset and duration of immunity induced by the canarypox-based EI vaccine were studied in ponies. A first set of ponies were experimentally infected with the pathogenic EIV strain A/eq/Kentucky/91, 2 weeks after a single immunisation with the canarypox-based vaccine. Vaccinated animals showed significantly reduced signs of disease when compared with control ponies. The amount of virus shed was also decreased but not its duration, which indicates that vaccinates could remained a source of virus and disease transmission [122]. Field results from the 2007 Australian outbreak report that frequency of infection, severity and duration of clinical signs of disease and duration of virus shedding were significantly reduced in vaccinated horses when exposed a few days after the first immunisation with the canarypox-based EI vaccine, when compared to unvaccinated horse population, supporting a rapid onset of immunity after only one immunisation [123]. An accelerated schedule of vaccination (only 14 days between the first and second immunisation) was tested during the 2007 Australian outbreak. Protective levels of SRH antibody were measured [124,125]. This accelerated schedule of vaccination was applied in several Australian states, such as New South Wales [126]. The accelerated immunisation schedules could prove extremely useful in an emergency situation, such in Australia. Protective levels of SRH antibody were measured up to 4 months after V3 following the accelerated schedule [125], but in the absence of a control group immunised according to the vaccine label, it is difficult to evaluate the potential impact of the accelerated schedule on the long-term duration of immunity (e.g., up to annual boost immunisation). Protection induced by the new fully updated canarypox-based EI vaccine when tested at the onset of immunity against the A/eq/Richmond/1/07 strains has been anecdotally reported by the vaccine manufacturer. 

A delayed SRH antibody response was reported after annual boost immunisation with the canarypox-EI vaccine when compared with other commercially EI vaccines and suggests that horses should preferably receive their booster immunisation no later than 4 weeks prior to an event [127] (study sponsored by the Irish Department of Agriculture). Duration of immunity was evaluated 5 and 6 months after completion of the primary course of vaccination (V1 and V2) or 1 year after the boosting immunisation (V3). Five and 6 months post V2, at the time of possible susceptibility to infection (immunity gap), vaccinated ponies were significantly protected against experimental infection with either the EIV strains A/eq/Sussex/89 or A/eq/Kentucky/91, respectively [122,128]. In both challenge studies, the level of virus shedding was also significantly decreased. However, duration of virus excretion remained unchanged after challenge with A/eq/Kentucky/91 [122]. Vaccinated ponies were significantly protected against infection with A/eq/Sussex/89, 1 year post boost immunisation (V3) [128] (study sponsored by Merial Ltd.). This long-term protection was probably based on a strong antibody response. One year post V3, the average level of SRH antibody was still around 100 mm² [128]. The antibody response is also seconded by IFN gamma and IL-2 responses, which support stimulation of CMI after immunisation [120,129,130] (studies sponsored by Merial Ltd.). The canarypox-based EI vaccine encodes only HA, which is not a major CTL target protein in humans. It is the conserved influenza NP and M proteins that are known to contain immunodominant CTL epitopes [131]. The importance of a HA-specific CMI and the EIV immunodominant CTL epitopes are unknown in the horse.

The presence of pre-existing immunity to the vaccine vector may reduce efficiency of subsequent immunisation with the same vector, as previously demonstrated in the case of recombinant vaccinia viruses [132]. In the horse, stimulation of cellular and/or humoral immune responses to the canarypox vector does not appear to impact efficiency of subsequent immunisation with the same or another canarypox-based vaccine [133]. Canarypox-based vaccines have been developed against West Nile virus and African Horse sickness [134], although the latter is not yet commercially available. Interestingly, the canarypox-HA vaccine has been tested in dogs and has been shown to stimulate a humoral immune response against canine influenza viruses (CIV) [135], which are related to EIV [136].

#### 3.5.2. Herpes Virus as Vaccine Vectors

Equine Herpes virus type 1 (EHV-1) has recently been used as vaccine vector for EIV immunisation. A recombinant EHV-1 (RacH strain) expressing HA from the EIV strain A/eq/Ohio/03 was generated and used to immunise dogs against CIV. Four dogs received two subcutaneous inoculations of the recombinant EHV-1 vaccine (rH-EIV), 4 weeks apart, and were experimentally infected with the CIV strain A/canine/PA/1095-07, 3 weeks after the second immunisation. An H3N8 influenza virus-specific antibody response was measured in dogs after vaccination. Vaccinated dogs showed reduced clinical signs of disease and virus shedding after experimental infection with a virulent strain of CIV [137]. A similar recombinant EHV-1 vaccine vector, based on the recent abortogenic EHV-1 strain NYO3 and expressing the HA from A/eq/Ohio/03, was used to immunise three adult horses, twice, 5 weeks apart. The EIV-specific antibody responses, measured by HI, were detectable from 2 weeks post V1 and up to 18 weeks post V2. Protection induced by the recombinant EHV-1 vaccine was not challenged by experimental infection with EIV [138]. The risk of EHV-1 latency establishment and reactivation has not been investigated.

### 3.6. Adverse Event to EI Immunisation

Vaccination corresponds to the injection of a foreign immunogenic substance to the host. Therefore, the aim of vaccination is to induce a protective immune response, though stimulation of the immune system. This could not be achieved without the activation of numerous physiological process, such an inflammatory response at the site of immunisation [96]. In this context, appearance of signs subsequent to immunisation (such as local oedema, mild pyrexia), in the hours or days following the vaccine administration is not necessarily abnormal, from an immunological point of view. However, these transient side effects should not exceed a few days. The incidence rate is considered as rare; with one reported case for every 1000–10,000 vaccination (France) (Agence Nationale du Médicament Veterinaire (ANMV) ANSES Pharmacovigilance Dpt Centre National d’Informations Toxicologiques Vétérinaires CNITV Lyon), but could be under evaluated due to the lack of reporting. 2/3 of adverse events are classified as mild or minor, such as a transient swelling (1–5 days) at the site of injection (<5 cm; reported in up to 25% of Australian horses vaccinated with the canarypoxvirus EI vaccine [139]), transient lethargy (<5% of horses in Australia), transient stiffness and swelling in the limbs (<1%). Serious adverse events usually represent 1/3 of reported cases (mostly anaphylactic shock). It has been suggested that hypersensitivity reaction to vaccination, such as anaphylactic shock, may involve the gradual development of an IgE-based antibody response and subsequent sensitivity to non-target components common to several equine vaccines, such as bovine serum albumin [140]. A case of fibrosarcoma was reported in Australia after EI immunisation [141].

### 3.7. Vaccination in the Face of an Outbreak

Vaccination in face of an EI outbreak could significantly reduce the size of an epidemic [142]. The use of the canarypox-based EI vaccine was a contributing factor for the successful control of the 2007 EI outbreak in Australia and facilitated a return to normal racing and breeding conditions [126,143,144]. The possibility to use an EIV NP-specific ELISA to differentiate infected animals from vaccinates (DIVA) and the effective use of this vaccine during the 2003 South African EI outbreaks were important element in the selection of an EI vaccine during this outbreak [145]. The EIV NP ELISA was further evaluated for used as DIVA assay. Recent results confirmed the possibility to use this NP ELISA but only discriminate horses immunised with the canarypox-based EI vaccine (encoding the EIV HA gene only) [146].

The importance and role played by emergency vaccination in Australia was argued, as the number of infected cases were already decreasing at the time of vaccination and when protective immunity was effective. However, the model of the EI Australian outbreak revealed that vaccination implementation; alongside biosecurity measures and movement restriction were effective for the eradication of EI in Australia. Vaccination rings of 1 to 3 km around infected area gave good results, depending on the vaccination capacity available [147]. Ring vaccination was also used to prevent EIV transmission to wild horse populations in New South Wales in order to prevent the establishment of EIV reservoir [148]. Obviously, the rate of EIV transmission/infection and the subsequent morbidity decrease with increase rate of vaccination. However, it is important to note that presence of subclinically infected vaccinated horses may complicate clinical surveillance, and subsequent localisation and delineation of the outbreak [149]. Twelve months after the last reported case of EI, Australia regained its EI-free status [150].

## 4. Inefficient or Suboptimal Response of Horses to Vaccination 

The reduced or absent response to vaccination is a well-recognised phenomenon in which a small percentage of the vaccinated population fails to mount and/or maintain an adequate immune response and therefore remains susceptible to disease. Analysis of post-race sample showed that up to 7.5% of Thoroughbred horses had no detectable levels of SRH antibody, despite mandatory EI vaccination [69]. Poor vaccine responders could be separated in two categories. The first would regroup horses that transiently failed to mount an immune response after their first EI immunisation. This has been well documented in several recent works. The proportion of poor responders in this category could be as high as 79% after first immunisation, depending on the type of EI vaccine used [79,82]. The lower frequency of poor responders was measured after immunisation with the carbopol-adjuvanted whole inactivated EI vaccine. This specific vaccine was also shown to induce the highest levels of SRH antibody when compared with other EI vaccines, which could provide an explanation for the lower percentage of poor responder reported after first immunisation [79,82]. The age at the time of first vaccination also seems to influence the response to immunisation [151], with reduced EI vaccine immunogenicity in weanling and yearlings when compared with older horses [151]. Antibody titres are usually back to normal levels after the second immunisation. The second category would regroup long-term poor responder (*i.e.*, individuals that repeatedly failed to mount or maintain adequate immunity). A recent study has shown that 6 months after an annual EI boost immunisation, only 7 out of 44 horses had a SRH antibody level >150 mm², 5 horses were below the protective 85 mm² threshold and one was seronegative. This last horse responded to boost vaccination but had undetectable antibody titre 3 months after boost immunisation [127]. Inefficiently vaccinated horses are usually partially protected against EI and present a subclinical form of the disease that is likely to go unnoticed. Importantly, these horses may shed large amount of infectious viruses for longer periods, and as such may be important in propagating the disease. In 2003, the EI outbreak in Newmarket revealed that vaccination >3 months previously was a risk factor for infection [60]. The Australian 2007 outbreak quarantine inquiry report [28] retrospectively identified poor vaccine responders in the quarantined population as the source of EIV that propagated to the wider Australian horse population. As with any vaccination campaign, a threshold level of protected individuals of 83%–94% is usually required for adequate herd immunity [152,153]. The minimum vaccine coverage necessary to prevent EI outbreak is also dependent on the mismatch between the vaccine and the circulating EIV strains [65]. Recent epidemiological surveys indicate that reported EI vaccine coverage could be around 70%–75% in the UK, with 4% of these animals reported to be last EI immunised more than a year previously [154,155]. The field reality could be significantly lower. Partial protection due to low vaccine response will reduce the overall herd immunity, but also favours influenza virus antigenic drift that can lead to vaccine breakdown in the future [156]. A metapopulation model of EI spread amongst Thoroughbred horses based on the 2003 Newmarket EI outbreak indicated that risk and size of an epidemic increased significantly if poor vaccine responders are concentrated in a small area close to the index case [142]. Equine influenza vaccination has been the subject of several mathematical models, which have been recently reviewed [157]. It is also important to note that exercise has been shown to decrease T-cell mediated immunity and to increase susceptibility of vaccinated horses to EIV infection, when compared to rested vaccinates [158]. Any increase in understanding of how the horse population responds to vaccination and the identification of reasons behind a poor response to vaccination is of potential benefit in the management of equine influenza. A field study is currently being conducted in Northern France to evaluate the frequency of poor vaccine responder and to identify some of the factors involved (Pronost S., Paillot R. and Legrand L. European Regional Development Fund, Equine Immunology at Animal Health Trust, LABEO Frank Duncombe, University of Caen Basse-Normandie, 2013–2015).

### 4.1. Immunity Gap and Interference of Pre-Existing Immunity

EIV-specific antibody levels induced by vaccination usually decrease significantly between the second and third immunisation [127], creating a possible window of susceptibility or immunity gap [159]. A recent study has shown that vaccinated horses experimentally infected with EIV 2–3 months after the second immunisation (*i.e.*, with low or negative SRH antibody level at the time of contact with EIV) could be infectious and infect naïve sentinels for several days [106]. Successful EIV transmission was also demonstrated in up to four vaccinated ponies with SRH antibody levels <60 mm² [160]. The possibility for EI vaccine to close the immunity gap is debatable. Immunity induced by the canarypox-based and the ISCOM-Matrix-adjuvanted EI vaccines have been shown to protect against challenge infection with A/eq/Sussex/89, A/eq/Kentucky/91 or A/eq/Kentucky/9/95, 5–6 months after the second immunisation [102,122,128]. Those studies showed significant reduction of clinical signs of disease and virus shedding, but vaccinated still shed virus for several days. Epidemiological data from field outbreaks (UK in 2003 and Ireland from 2007 to 2010) clearly indicated an increased risk of infection in animals that had not received a boost immunisation in the 3 to 6 months prior to the outbreaks, irrespective of the EI vaccine used [23,61]. An accelerated schedule of vaccination was attempted in order to reduce susceptibility prior to boost vaccination. Thoroughbreds were immunized with a whole inactivated or an ISCOM-Matrix-adjuvanted EIV vaccine according to the recommended vaccination schedule or with an early third dose (8 weeks post V2 instead of 24 weeks). The level of antibody was lower after the accelerated vaccination schedule when compared with conventionally immunised horses. This was explained by the interference of pre-existing EIV-specific antibody at the time of the third boost immunisation [161] (study sponsored by Intervet Int.). Gildea *et al*. have recently reported that pre-existing SRH levels significantly correlate with the absence of response to boost immunisation [127]. These studies suggest a fine balance between partial immunity and immune interference at the time of the boost immunisation, which is dependent on the individual’s response to vaccination and supports a monitoring of the antibody response prior to immunisation. Three primary vaccination regimes were also recently tested (*i.e.*, minimum intervals permitted by racing authorities, product labels recommended and longest intervals allowed). Results from this study indicated that overall levels of SRH antibody reached at peaks of immunity were not significantly affected by the nature of the vaccination regimes, however, the duration of the period of susceptibility between V1 and V2, and subsequently V2 and V3 were significantly lengthened when the longest interval scheduled was applied [151] (study sponsored by the Irish Department of Agriculture). This study confirms that product’s label recommendations should be preferred, when possible. 

### 4.2. Immunisation with Multiple Different Types of Vaccines

During its lifetime, a race horse must be repeatedly vaccinated against equine influenza in order to travel internationally and to compete. However, the use of the same product throughout its lifetime/career is unlikely, due to change of owners, of veterinary practitioner and/or of geographic location [159]. The compatibility between commercially available vaccines against equine influenza has rarely been documented and remains a concern to veterinary practitioners. Intervet International reported that a heterologous vaccination regime consisting of a primary immunisation with the Flu Avert IN vaccine (live attenuated virus vaccine) followed by boost immunisation with Equilis Prequenza (ISCOM-Matrix sub-unit vaccine), 4 weeks later, induced stronger immunity than a homologous vaccination (prime/boost immunisation with Flu Avert IN alone) and provided sterile immunity when experimentally challenged with EIV, 3 weeks after the last vaccination [162]. Regretfully, the data displayed in this patent is limited. In contrast, the influenza outbreak affecting Newmarket (UK) in 2003 has been extensively studied and data analyses also support the beneficial short term impact of mixing vaccine technologies [61]. This epidemiological study reported that horses that were vaccinated with more than one type of vaccine were at lower risk of developing EI. Protection was also improved if the last vaccine used was not an inactivated vaccine containing whole EIV and aluminium hydroxide as adjuvant. The beneficial impact of mixed vaccination reported in this study may be explained by a low efficiency of the inactivated EIV, aluminium hydroxide based vaccine used at the time of this outbreak. Immunity to EIV was apparently enhanced by the use of any other type of vaccine. Compatibility of EI vaccines will require further investigation.

### 4.3. Vaccination with Multiple Different Virus Strains—Effect of Antigenic Distance

Horses are relatively long lived and are likely to received repeated immunisations against EI. Equine influenza vaccines from different manufacturers often contain or express antigens from different strains of EIV (e.g., A/eq/Newmarket/1/93, A/eq/Kentucky/98 and A/eq/Ohio/03 as representative strains of the American lineage). Even if a unique brand of vaccine is used, immunisation with different EIV strains will also occur “naturally” over a horse life-span as vaccines are updated. This diversity may influence the quality and specificity of the immune response after boost immunisation, due to antigenic variation between the isolates used. It has been demonstrated that strain-specific antibody to influenza is more effective than cross-reactive antibody [163]. Post vaccination equine sera are generally considered as being highly cross-reactive [164,165,166] rather than strain-specific; vaccination with multiple different strains may increase this tendency. Changing the strains in EIV vaccines may also favour the theoretical mechanism of original antigenic sin (OAS) [167]. OAS is characterised by the production of antibody that shows higher affinity to viral epitopes shared with a previously encountered influenza virus strain rather than the virus strain present in the most recent vaccine. The OAS involvement in EIV immune response remains controversial [61,168,169].

### 4.4. Effect of Age and Maternally Derived Antibody (MDA) on the Immune Response to EI Vaccination

The level of MDA in foals could be elevated in the first months of age, especially if the mare is vaccinated and/or boost immunised in the last months of gestation, as recently demonstrated [170]. Primary EI vaccination often occurs around 5 to 6 months of age, depending on the vaccine used, when the level of MDA is low enough to avoid any interference, and whether the foal’s immune response is mature enough for efficient antigen priming. The effect of maternally derived antibodies (MDA) at the time of first immunisation with the canarypox-based EI vaccine has been studied. Foals (10 to 20 weeks old) born to EI vaccinated mares received a first vaccine dose in presence of MDA and 2 others once MDA had declined. Canarypox-based EI immunisation in presence of MDA did not result in measurable production of SRH antibodies. However, the immune response may have been primed; the SRH antibody levels were transiently higher after boost immunisation (V2) when compared with foals of a similar age that had received only one canarypox-based EI immunisation [171] (study sponsored by Merial Ltd.). It has also been reported that the canarypox-based EI vaccine could be used in foals as young as 1 day of age [124]. However, the amount of data presented was limited.

On the other hand, aged horses tend to display impaired response to EI vaccination [172]. Twenty to 28 year old horses immunised with the canarypox-based EI vaccine remained susceptible to EIV infection with A/eq/Kentucky/5/02 despite evidence of both humoral and cell-mediated immunity at the time of infection [130]. The overall clinical signs of disease and virus shedding were decreased when compared with non-vaccinated horses. However, their immune response to vaccination and/or challenge was significantly lower than naïve young animals (7 to 10 months old) [130]. Another group reported that younger animals had significantly higher titres of EI-specific antibodies after immunisation than aged horses [173]. These data are consistent with the 10-fold decrease of the EIV-specific antibody levels previously reported in aged horses, when compared with young animals [174]. This impaired immune response observed in aged horses is attributed to an overall decline of both T and B lymphocyte subsets [175]. 

## 5. Conclusions

Equine influenza is a highly infectious disease that can rapidly spread and induce high morbidity in susceptible horse populations. The impact of EI outbreaks on the equine industry can be significant. For these reasons, EI remains a major concern for owners, and veterinary practitioners worldwide. Alongside EI surveillance, quarantine and isolation of affected animals, vaccination represents one of the most important tools to prevent or contain EIV infection. Several efficient EI vaccines are available. A recent comparative study reported little difference of the SRH antibody response, the absolute correlate of protection against EIV, measured in a limited number of animals after immunisation with the different types of EI vaccines commercialised in Europe [127]. Results from cross-protection studies also indicate that the majority of EI vaccines commercially available would provide a good level of protection if used in the face of an imminent outbreak, when boost immunisation and overall increase of herd immunity is essential. However, the frequency of EI outbreaks in recent years, the continuous antigenic variation of EIV and the occasional cases of vaccine breakdown clearly indicate that a strong EI surveillance has to be maintained. An epidemiological survey conducted in Ireland from 2010 to 2012 indicates that EI vaccines outbreaks reported occurred with 4 different EI vaccines commercially available, irrespective of their strain composition [45]. Vaccination records from these individuals tend to indicate that infection occurred 5 months or more since last immunisation, when protective immunity is fading (e.g., immunity gap between V2 and V3). While pre-existing SRH antibody titres are still a strong correlate of protection, the protection threshold (e.g., 85 and 150 mm²) may have changed due to strain mismatch [45]. In these conditions, the necessity to update the EIV strains currently contained in commercialised vaccines is increasingly pressing. The diversity of EI vaccine technologies currently commercialised represents an asset to the equine industry as it would probably minimise the risk and impact of generalised vaccine breakdown. Numerous studies are currently conducted in human research to design an efficient universal influenza vaccine that would overcome difficulties associated with antigenic drift and shift. Several approaches are being evaluated (e.g., multivalent HA vaccine, highly conserved stalk epitopes, conserved target proteins such as NP and M2, T-cell vaccine, *etc*.) and results from these research projects will undeniably influence the design of future EI vaccines. Until then, a better understanding of the factors that influence vaccination efficiency, improvement of the vaccine coverage, more frequent immunisation, identification of poor vaccine responders and a targeted monitoring of the antibody response prior to immunisation, as recently suggested, would benefit overall protection against EI.
